# Retinal Vasculopathy in Alzheimer’s Disease

**DOI:** 10.3389/fnins.2021.731614

**Published:** 2021-09-22

**Authors:** Haoshen Shi, Yosef Koronyo, Altan Rentsendorj, Dieu-Trang Fuchs, Julia Sheyn, Keith L. Black, Nazanin Mirzaei, Maya Koronyo-Hamaoui

**Affiliations:** ^1^Department of Neurosurgery, Maxine Dunitz Neurosurgical Research Institute, Cedars-Sinai Medical Center, Los Angeles, CA, United States; ^2^Department of Biomedical Sciences, Cedars-Sinai Medical Center, Los Angeles, CA, United States

**Keywords:** cerebral amyloid angiopathy, vascular amyloidosis, eye, ocular disease, retinal imaging, blood retinal barrier, Alzheimer’s disease, neurodegenerative disease

## Abstract

The retina has been increasingly investigated as a site of Alzheimer’s disease (AD) manifestation for over a decade. Early reports documented degeneration of retinal ganglion cells and their axonal projections. Our group provided the first evidence of the key pathological hallmarks of AD, amyloid β-protein (Aβ) plaques including vascular Aβ deposits, in the retina of AD and mild cognitively impaired (MCI) patients. Subsequent studies validated these findings and further identified electroretinography and vision deficits, retinal (p)tau and inflammation, intracellular Aβ accumulation, and retinal ganglion cell-subtype degeneration surrounding Aβ plaques in these patients. Our data suggest that the brain and retina follow a similar trajectory during AD progression, probably due to their common embryonic origin and anatomical proximity. However, the retina is the only CNS organ feasible for direct, repeated, and non-invasive ophthalmic examination with ultra-high spatial resolution and sensitivity. Neurovascular unit integrity is key to maintaining normal CNS function and cerebral vascular abnormalities are increasingly recognized as early and pivotal factors driving cognitive impairment in AD. Likewise, retinal vascular abnormalities such as changes in vessel density and fractal dimensions, blood flow, foveal avascular zone, curvature tortuosity, and arteriole-to-venule ratio were described in AD patients including early-stage cases. A rapidly growing number of reports have suggested that cerebral and retinal vasculopathy are tightly associated with cognitive deficits in AD patients and animal models. Importantly, we recently identified early and progressive deficiency in retinal vascular platelet-derived growth factor receptor-β (PDGFRβ) expression and pericyte loss that were associated with retinal vascular amyloidosis and cerebral amyloid angiopathy in MCI and AD patients. Other studies utilizing optical coherence tomography (OCT), retinal amyloid-fluorescence imaging and retinal hyperspectral imaging have made significant progress in visualizing and quantifying AD pathology through the retina. With new advances in OCT angiography, OCT leakage, scanning laser microscopy, fluorescein angiography and adaptive optics imaging, future studies focusing on retinal vascular AD pathologies could transform non-invasive pre-clinical AD diagnosis and monitoring.

## Introduction

Alzheimer’s disease (AD) is the leading cause of senile dementia, accounting for 60–80% of total cases (Alzheimer’s Association, 2020). By 2050, over six million Americans are projected to live with AD, which could lead to a staggering $355 billion national financial burden (National Institue on Aging, 2019; Alzheimer’s Association, 2020). AD patients progressively develop irreversible cognitive loss due to neurodegeneration in the brain and other direct or indirect factors such as accumulation of toxic molecules, neuroinflammation, and vascular damage. The main pathological hallmarks of AD are amyloid β-protein (Aβ) accumulation and neurofibrillary tangles, mainly composed of hyperphosphorylated (p)tau deposits, that may exist inside or outside of neurons and in blood vessels (Bloom, 2014; Cisternas et al., 2019). Our group identified these hallmarks in the retina of postmortem and living AD and mild cognitively impaired (MCI) patients (Koronyo-Hamaoui et al., 2011; La Morgia et al., 2016; Koronyo et al., 2017). Investigation of CNS and fluid biomarkers has become an essential part of AD research. In 2018, the National Institute on Aging and Alzheimer’s Association (NIA-AA) created an updated research framework for classifying pathological phases of AD based on detection of abnormal levels of molecular biomarkers Aβ (A), tau (T), and neurodegeneration [AT(N)], regardless of cognitive status in living patients (Jack et al., 2018). The ATN framework was also proposed to be expandable to include new AD biomarkers such as vascular biomarkers (ATNV) (Jack et al., 2018).

Vascular pathology in AD is an expanding subject and a growing number of studies show that vascular-related damage in the brain and retina can predict cognitive decline (Vidal and Mavet, 1989; Baker et al., 2007; Gharbiya et al., 2014; Boyle et al., 2015; Bulut et al., 2016, 2018; Cunha et al., 2017; McGrory et al., 2017; Planton et al., 2017; Cabrera DeBuc et al., 2018; Deal et al., 2018; Jiang et al., 2018; O’Bryhim et al., 2018; van der Flier et al., 2018; Iadecola et al., 2019; Jung et al., 2019; Montagne et al., 2020; Shi et al., 2020a; Li et al., 2021). Cerebral vascular damage such as ischemia leads to disturbed nutrient supply, induces oxidative stress and inflammatory activities, impedes Aβ clearance and/or alters amyloid-processing enzymes (Marchesi, 2011), all of which can contribute to neurodegeneration and cognitive decline. Studies have also proposed that the onset of clinical dementia may be preceded by reduced cerebral blood flow associated with insufficient Aβ clearance (Wolters et al., 2017; Govindpani et al., 2019). With new disease-modifying therapies on the horizon and emphasizing the need for early intervention (Tonda-Turo et al., 2018), the current challenge is to diagnose AD early and accurately in the clinical setting to allow for an effective outcome that could limit the damage and prevent further disease progression.

### Vascular Damage in AD Brain

The brain is nourished by one of the human body’s richest networks of blood vessels (Prensa, 2014), rendering its vascular network highly susceptible to aging and AD-related cerebral damage. Studies indicate that AD pathology is associated with severe effects on cerebral blood vessels, potentially by a wide range of complications (Govindpani et al., 2019). These include cerebral amyloid angiopathy (CAA) (Ellis et al., 1996; Arvanitakis et al., 2011; Viswanathan and Greenberg, 2011), vascular non-perfusion (Bonte et al., 1986; Hirsch et al., 1997; Binnewijzend et al., 2016), neurovascular unit (NVU) uncoupling and degeneration (Higuchi et al., 1987; Vinters et al., 1994; Claudio, 1996), angiogenesis (Desai et al., 2009; Biron et al., 2011), small blood vessel distortions (Hassler, 1965; Beskow et al., 1971; Fischer et al., 1990; Kalaria and Kroon, 1992), blood–brain barrier (BBB) breakdown and damage (Slemmon et al., 1994; Zipser et al., 2007; Bell and Zlokovic, 2009; Ryu and McLarnon, 2009; Sengillo et al., 2013; van de Haar et al., 2016a, b), vascular tau accumulation (Williams et al., 2005; Castillo-Carranza et al., 2017), dysregulated glucose metabolism (Kalaria and Harik, 1989; Harik, 1992), inflammation (Grammas and Ovase, 2001; Tripathy et al., 2007), hypertension (Launer, 2002; Gabin et al., 2017), hypercholesterolemia (Matsuzaki et al., 2011), and atherosclerosis (Alzheimer, 1911; Yarchoan et al., 2012).

Amyloid plaques are the most considerable hallmarks of AD, with 42 and 40 amino acid-long Aβ alloforms tightly associated with AD pathogenesis and vascular pathology (Blennow et al., 2015; Selkoe and Hardy, 2016). Nearly 85% of AD patients develop varying degrees of CAA complications (Arvanitakis et al., 2011; Viswanathan and Greenberg, 2011), defined by Aβ deposits inside walls of arteries, arterioles and capillaries (DeSimone et al., 2017). Accumulation of Aβ within blood vessels is associated with damage to muscular and elastic tissue, possibly replaced by Aβ fibrils, leading to lobar cerebral hemorrhage (ICH) or vascular non-perfusion (Mehndiratta et al., 2012; Keable et al., 2016). CAA can also trigger other pathogenic pathways, such as inflammation and oxidative stress, further leading to cerebral tissue damage (Ghiso et al., 2010).

### Alzheimer’s Retinopathy

Over the past decade, the retina has been extensively investigated as a top candidate site of AD manifestation beyond the brain, as it shares many structural, cellular, molecular, and functional similarities with the brain (Hinton et al., 1986; Purves, 2001; Patton et al., 2005; Koronyo-Hamaoui et al., 2011; Koronyo et al., 2012, 2017; Schon et al., 2012; Erskine and Herrera, 2014; Crair and Mason, 2016; Hart et al., 2016; La Morgia et al., 2016; den Haan et al., 2018a; Asanad et al., 2019; Grimaldi et al., 2019; Lee S. et al., 2020; Mirzaei et al., 2020; Schultz et al., 2020; Snyder et al., 2021). Given the parallel pathology in the brain and retina, the retina has the potential to become a non-invasive diagnostic window since it is not shielded by bone and is easily accessible by ophthalmic exams such as optical coherence tomography (OCT) and fundoscopy (including scanning laser ophthalmoscopy) with subcellular resolution. The retina is directly and indirectly connected to the brain through bundles of neuronal axons forming the optic nerve, and by retinal and cerebral blood vessels, which may facilitate transportation of abnormal Aβ and tau species and further lead to the spread of AD pathology throughout the CNS (Morin et al., 1993). In addition, the discovery of dysfunctional lymphatic vessels within the brain of rodent models of AD implicates this CNS-specific lymphatic network, referred to as the glymphatic system (Jessen et al., 2015), as a culprit of insufficient cerebral amyloid clearance in AD (Louveau et al., 2015; Da Mesquita et al., 2018; Ahn et al., 2019). Recently, an ocular lymphatic drainage system was also identified in rodent models, which relies on an aquaporin-4-dependent pathway to clear fluid and metabolites (Wang et al., 2020). The roles of such lymphatic systems in retinal diseases and AD remain to be explored in future studies.

Studies conducted by OCT, electroretinogram (ERG), and histological examinations on cognitively impaired patients and laboratory animals have extensively described various retinal pathological and functional changes associated with AD development. In fact, the retina is heavily affected by AD pathology and displays a wide spectrum of retinopathy (reviewed in Mirzaei et al., 2020). This includes optic nerve degeneration and retinal neuronal and ganglion cell (RGC) loss (Hinton et al., 1986; Blanks et al., 1989, 1996; La Morgia et al., 2016; Koronyo et al., 2017; Asanad et al., 2019), retinal nerve fiber layer (NFL) thinning (Kergoat et al., 2001; Parisi et al., 2001; Berisha et al., 2007; Paquet et al., 2007; Moschos et al., 2012; Kirbas et al., 2013; Marziani et al., 2013; Moreno-Ramos et al., 2013; Kromer et al., 2014; Shi et al., 2014; Bayhan et al., 2015; Coppola et al., 2015; Gao et al., 2015; Liu et al., 2015; La Morgia et al., 2016), gliosis (Hinton et al., 1986; Curcio and Drucker, 1993; Blanks et al., 1996; Guo et al., 2010; Grimaldi et al., 2019), and vascular degeneration and injury (Patton et al., 2005; Frost et al., 2013; Cheung et al., 2014; Feke et al., 2015; Williams et al., 2015; Kapasi and Schneider, 2016; Shi et al., 2020b). This retinal damage can explain, at least in part, the visual dysfunctions (Sadun and Bassi, 1990; Armstrong and Syed, 1996; Risacher et al., 2020), sleep disturbances (La Morgia et al., 2016; Wang and Holtzman, 2020), and ERG abnormalities (Trick et al., 1989; Parisi et al., 2001; Moschos et al., 2012) documented in AD patients. Such findings have largely encouraged basic research in the AD retina and exploration of retinal imaging techniques for AD diagnosis.

Our group was the first to demonstrate the existence of Aβ accumulation, the hallmark AD pathology, in the retina of AD patients, including early-stage cases. In a study published in mid-2010, we revealed the aggregation of Aβ deposits in retinal flat-mounts isolated from 13 out of 13 neuropathologically confirmed AD and mild cognitively impaired (MCI) patients, which was minimally or undetected in 5 cognitively normal (CN) subjects negative for brain amyloid (Koronyo-Hamaoui et al., 2011). Further, this pioneer study demonstrated for the first time the ability to non-invasively detect curcumin-labeled Aβ deposits in live murine models of AD (Koronyo-Hamaoui et al., 2011). Importantly, similar reductions in retinal and brain Aβ plaques were detected *ex vivo* and *in vivo* in AD-model mice (Koronyo-Hamaoui et al., 2011; Koronyo et al., 2012) in response to immunomodulation therapies (Butovsky et al., 2006; Koronyo-Hamaoui et al., 2009; Bakalash et al., 2011; Koronyo et al., 2015; Rentsendorj et al., 2018; Doustar et al., 2020). Although a few studies failed to detect Aβ and/or (p)tau in the retina of AD patients, these reports included low case numbers (Schon et al., 2012; Ho et al., 2014; Williams et al., 2017) and only examined limited retinal regions in cross sections, focusing on less affected regions in these patients (La Morgia et al., 2016; Koronyo et al., 2017; Asanad et al., 2019; Shi et al., 2020b). It is possible this discrepancy in findings could also be due to differences in retinal tissue preservation, processing, and/or immunostaining protocols.

Subsequent studies by La Morgia et al. (2016), Lee S. et al. (2020), and others also demonstrated Aβ plaques and vascular-associated deposits in postmortem retinas of AD patient cohorts. Retinal amyloidosis in AD patients was in stark contrast to minimal pathology observed in the retinas of CN individuals (Tsai et al., 2014; La Morgia et al., 2016; den Haan et al., 2018a; Grimaldi et al., 2019; Lee S. et al., 2020; Qiu et al., 2020; Shi et al., 2020a; Cao et al., 2021). In 2017, Koronyo et al. (2017) published the development of more advanced human retinal extraction and histological techniques. Authors utilized immunofluorescence, anti-Aβ compound labeling, non-fluorescence immunostaining, and transmission electron microscopy (TEM) to measure Aβ_42_ plaque burden, characterize retinal Aβ plaque subtypes and morphology including identifying retinal Aβ fibrils and protofibrils, and describe Aβ plaque topographical and layer distribution in a larger cohort of 23 AD patients vs. 14 age- and sex-matched CN patients (Koronyo et al., 2017). In this study, several Aβ-epitope labeling techniques including Gallyas silver stain, curcumin, thioflavin-S, congo red, as well as a combination of monoclonal antibodies against various N’-, C’- and center Aβ sequences were used to describe amyloidosis in the human AD retina. Hence, together with post-mortem detection by immunofluorescence staining, peroxidase-based staining, and TEM analysis on retinal flat-mounts and cross-sections, this study profoundly validated Aβ accumulation in the AD retina in comparison to CN controls. We also demonstrated a significant correlation between retinal and brain plaque burdens, and more importantly, provided the first proof-of-concept trial using curcumin labeling and a scanning laser ophthalmoscope to detect and quantify retinal Aβ plaques in living patients, (Koronyo et al., 2017).

Indeed, multiple biochemical and histological studies corroborated these findings of Aβ deposits in the human AD retina (den Haan et al., 2018a; Grimaldi et al., 2019; Lee S. et al., 2020; Qiu et al., 2020) and further described retinal pTau, Aβ_40_ and Aβ_42_ accumulation, inflammation, and correlations between retinal and cerebral Aβ levels in AD patients (Alexandrov et al., 2011; Schon et al., 2012; den Haan et al., 2018b; Grimaldi et al., 2019; Lee S. et al., 2020; Qiu et al., 2020; Schultz et al., 2020; Shi et al., 2020b). More recently, *in vivo* retinal amyloid imaging in living MCI and AD patients was achieved via either retinal curcumin-enhanced fluorescence and SLO imaging or hyperspectral imaging (Hadoux et al., 2019; More et al., 2019; Dumitrascu et al., 2020; Lemmens et al., 2020; Ngolab et al., 2021).

Recent studies by Chibhabha et al. (2020); Sidiqi et al. (2020), and Barton et al. (2021) in the APP_SWE_/PS1_Δ*E*9_ transgenic mouse model further corroborated these findings via Aβ retinal curcumin imaging. In fact, numerous studies in AD rodent models have detected Aβ and its alloforms such as Aβ_40_ and Aβ_42_ in the AD retina (Inestrosa et al., 2005; Dutescu et al., 2009; Liu et al., 2009; Alexandrov et al., 2011; Ardiles et al., 2012; Schon et al., 2012; Williams et al., 2013; Yang et al., 2013; Zhao et al., 2013; Park et al., 2014; Tsai et al., 2014; Du et al., 2015; Parthasarathy et al., 2015; Chiasseu et al., 2017; Grimaldi et al., 2018; Harrison et al., 2019).

### Retinal Vascular Aβ Deposits in AD Patients and Animal Models

An early study by Liu et al. (2009) in the Tg2576 transgenic murine model describes Aβ deposits within retinal microvessels by immunostaining against various Aβ epitopes, using mAbs clones 6E10, 12F4 and 5C3, in retinal cross-sections. Histological examinations by La Morgia et al. (2016) and Koronyo et al. (2017) of retinas from AD patients and age- and sex-matched cognitively normal controls provided evidence for retinal Aβ deposits inside blood vessel walls, perivascular and along blood vessels by immunostaining for 12F4-positive Aβ_42_ in retinal flat-mounts and cross-sections. In the Koronyo et al. (2017) study, retinal vascular Aβ accumulation in retinal flat-mounts and cross-sections of AD patients was also validated by other techniques including congo red, Gallyas silver stain, curcumin, 11A50-B10-positive Aβ_40_ immunostaining, as well as TEM analysis (Koronyo et al., 2017). In murine models of AD, a study by the same team demonstrated that following systemic administration of curcumin to APP_SWE_PS1_ΔE9_ model mice, *ex vivo* examination of retinal flatmounts revealed double-labeling of curcumin with 4G8 for Aβ deposits inside retinal blood vessels (Koronyo-Hamaoui et al., 2011).

**FIGURE 1:**
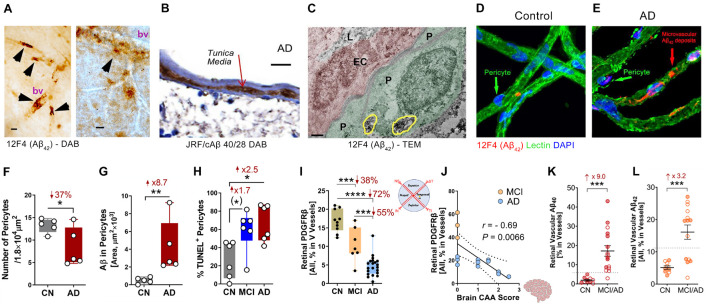
Retinal vascular amyloidosis and pericyte loss in the retina of MCI and AD patients. **(A)** 3,3′-Diaminobenzidine (DAB) staining of Aβ_42_ by 12F4 antibody in retinal blood vessels from flat-mount retina in an AD patient. Scale bar = 20 μm. **(B)** DAB staining of Aβ_40_ by JRF/cAβ 40/28 antibody on a retinal cross-section sample from an AD patient. Scale bar = 20 μm. **(C)** Transmission electron microscopy (TEM) for Aβ_42_ by 12F4 antibody staining in retinal blood vessels and pericytes. P, pericyte; EC, endothelial cell; L, lumen. Yellow circles indicate Aβ_42_ staining. Scale bar = 0.5 μm. **(D,E)** Immunostaining of Aβ_42_ by 12F4 antibody on retinal blood vessels isolated from an AD patient and control. Scale bars = 20 μm. **(F)** Quantification of pericytes in AD patients and cognitively normal (CN) controls based on isolated blood vessels. **(G)** Stereological quantification of Aβ in pericytes in AD patients and CN controls based on isolated blood vessels. **(H)** Quantification of terminal deoxynucleotidyl transferase-mediated dUTP nick-end labeling (TUNEL) positive pericytes on retinal cross-sections from CN, mild cognitively impaired (MCI), and AD patients. **(I)** Stereological quantification of PDGFRβ on retinal cross-sections from CN, MCI, and AD patients. **(J)** Pearson’s (r) correlation between cerebral amyloidosis angiopathy (CAA) and retinal PDGFRβ from MCI and AD patients. **(K,L)** Stereological quantification of panel **(K)**. Aβ_40_ and **(L)** Aβ_42_ in CN versus MCI/AD patients. Filled circles represent males and clear circles represent females. Data from individual human donor as well as groups are shown as mean ± SEM. *p < 0.05, **p < 0.01, ***p < 0.001, ****p < 0.0001, by one-way ANOVA with Sidak’s *post hoc* multiple comparison test (more than 2 groups) or unpaired 2-tailed Student’s t test (2 groups). Fold and percentage changes are shown in red. Panel A reproduced from Koronyo et al. (2017) with permission of ASCI via Copyright Clearance Center. Panels B–L reproduced from Shi et al. (2020b) under terms of the Creative Commons Attribution 4.0 International License (http://creativecommons.org/licenses/by/4.0/).

Amyloidosis in cerebral blood vessels predominately consists of Aβ_40_ alloforms (Gravina et al., 1995). Accordingly, Shi et al. (2020b) conducted the first stereological quantification and mapping of Aβ_40_ in retinal blood vessels by immunostaining of 11A50-B10 and JRF/cAβ 40/28—specific monoclonal antibodies detecting the Aβ_40_ alloform—in retinal cross-sections and isolated retinal blood vessels from MCI and AD patients (see Figures 1A–E,G,K,L for retinal vascular amyloidosis). The pattern that was revealed by Aβ_40_ immunoreactivity covered most vascular compartments including tunica media, adventitia, and intima, indicating retinal blood vessels may also be thoroughly affected by Aβ deposition (Figure 1B). Increased levels of Aβ_1–40_ peptides in the retina of AD patients as compared with age- and sex-matched cognitively normal controls was further validated by a sandwich enzyme-linked immunosorbent (ELISA) analytical biochemistry assay (Shi et al., 2020b). When correlated with cerebral pathologies, levels of retinal Aβ_40_ significantly associated with entorhinal cortex plaque load and had a trend of predicting cognitive decline and CAA. Retinal vascular Aβ_40_ tightly associated with neuritic plaques in the entorhinal cortex and combined cerebral regions including hippocampus, frontal cortex, temporal cortex, and parietal cortex. A study by Schultz et al. (2020) also successfully correlated levels of retinal high molecular weight Aβ_42_ and Aβ_40_ with neurofibrillary tangles (NFT) and Aβ scores in the hippocampus of AD patients. Another notable finding was the downregulation of low-density lipoprotein receptor-related protein 1 (LRP1) in AD retina, suggesting compromised Aβ clearance (Shi et al., 2020b).

In a subsequent report, Shi et al. (2020a) detected Aβ_40_ accumulation in retinal blood vessels of 8-month-old APP_SWE_PS1_ΔE9_ mice. Another recent study by Habiba et al. (2021) revealed detectable levels of Aβ_40_ and Aβ_42_ oligomers in the retina and blood as early as in 3-month-old APP/PS1 mice, prior to their detection in the respective brain. It is important to note that the transgenic APP/PS1 mouse model is driven by increased production of human amyloidogenic Aβ peptides, and therefore does not fully represent the human disease. Nevertheless, this mouse model is known to develop Aβ plaques and intracellular soluble Aβ oligomers, (p)tau, pronounced micro- and astrogliosis, synaptic loss, as well as cognitive and visual decline (Jankowsky et al., 2003; Butovsky et al., 2006; Koronyo-Hamaoui et al., 2009; Bakalash et al., 2011; Koronyo et al., 2015; Rentsendorj et al., 2018; Doustar et al., 2020; Vit et al., 2021). Intriguingly, a recent study by Chintapaludi et al. (2020) detected early onset alterations of retinal inflammatory genes before cerebral amyloidosis. Nevertheless, more supporting evidence and validation is needed to further evaluate the feasibility to diagnose AD by retinal vascular amyloid imaging.

### AD-Related Retinal Vasculopathy

Mounting evidence has demonstrated a wide range of retinal vascular abnormalities in both AD patients and animals, such as reduced macular microvascular density (O’Bryhim et al., 2018), decreased blood flow (Berisha et al., 2007; Feke et al., 2015; Einarsdottir et al., 2016), compromised microvascular network (Frost et al., 2013; Cheung et al., 2014; Williams et al., 2015; Einarsdottir et al., 2016; Cabrera DeBuc et al., 2018), damaged vascular branching complexity (Frost et al., 2013; Cheung et al., 2014), vein narrowing (Berisha et al., 2007; Frost et al., 2013; Cheung et al., 2014; Feke et al., 2015; Cabrera DeBuc et al., 2018), and increased vascular tortuosity (Cheung et al., 2014). Among these findings, several studies showed significant correlations between retinal vascular impairment and AD susceptibility, while others did not. Nevertheless, these discoveries have provided numerous potential retinal vascular targets for AD monitoring and diagnosis. Compared to the brain, a distinct feature of the retina is the existence of Müller glial cells, which are the principal retinal glial cell type that maintain neuronal activity by regulating extracellular concentration of neurotransmitters and neuroactive ions (Newman and Reichenbach, 1996). Indeed, a previously published report suggested that retinal Aβ is engulfed by these specialized Muller glial cells (den Haan et al., 2018b), warranting further research on the potential role of these retina-specific glial cells in AD pathogenesis. It is important to note that most investigations are still limited to cross-sectional observations. Future studies should seek to apply standardized protocols and design with longitudinal study methods.

Another similarity between the retina and brain is the blood-organ barrier: the blood–retinal barrier (BRB) is highly comparable to the BBB, both structurally and functionally (Campbell and Humphries, 2012; Zenaro et al., 2017; Cai et al., 2018). The BBB is composed of cerebral vascular endothelial cells with tight junctions (TJ), astrocyte end-feet and supporting pericytes, while the BRB is made of an inner barrier of retinal vascular endothelial cells and an outer barrier of retinal epithelial cells, both with TJ and supporting pericytes (Campbell and Humphries, 2012; Zenaro et al., 2017; Cai et al., 2018). The main functions of these barriers are to modulate the influx of ions, proteins and water, as well as curb the infiltration of circulating immune cells (Cunha-Vaz et al., 2011). In AD, a compromised BBB is viewed as one of the principal causes for cerebral amyloidosis due to its essential role in clearing abundant cerebral Aβ to the circulating blood via the vascular network (Zlokovic et al., 1993; DeMattos et al., 2002; Banks et al., 2003; Do et al., 2015; Zhao et al., 2015; Sweeney et al., 2018). Recently, the Zlokovic group has successfully connected the BBB-associated pericyte injury biomarker, soluble PDGFRβ, in cerebrospinal fluid (CSF) to cognitive decline in apolipoprotein E (APOE4) carriers even after controlling for Aβ and tau status (Montagne et al., 2020). These findings suggest that BBB biomarkers might be an option for next-generation AD diagnostics and therapeutics.

**FIGURE 2:**
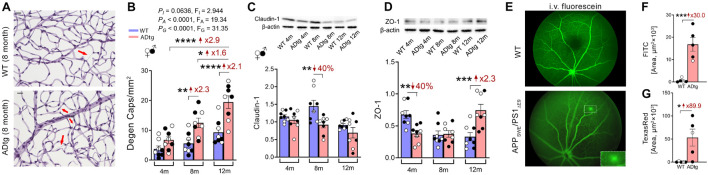
Retinal vasculopathy in APP_SWE_PS1_ΔE9_ (ADtg) mice. **(A,B)** Representative images of periodic acid-Schiff (PAS)-stained, hematoxylin-counterstained isolated retinal microvasculature from ADtg and matched wild type (WT) littermates. Acellular degenerated retinal capillaries are indicated by red arrows. **(B)** Numbers of degenerated retinal capillaries when mice are stratified by mouse genotypes, WT or ADtg, by age groups of 4, 8, and 12 months. **(C,D)** Western-Blot analysis of panel **(C)** claudin-1 and **(D)** ZO-1 in retinal lysates from 4, 8, and 12-month-old APP_SWE_PS1_Δ E9_ mice and WT controls. **(E)** Images showing *in vivo* retinal microvascular imaging for leakage after intraperitoneal fluorescein injection in 12-month-old WT and ADtg mice. **(F,G)** Quantitative analysis of the panel **(D)** FITC (1,000 kDa) or **(E)** Texas Red (3 kDa)-stained area in retinal flat-mounts from WT or ADtg mice. Black-filled circles represent males and clear circles represent females. Data from individual mouse as well as groups are shown as mean ± SEM. *p < 0.05, **p < 0.01, ***p < 0.001, ****p < 0.0001, by 2-way ANOVA with Sidak’s *post hoc* multiple comparison test (more than 2 groups) or unpaired 2-tailed Student’s t test (2 groups). Fold and percentage changes are shown in red. Reproduced from Shi et al. (2020a) under terms of the Creative Commons Attribution 4.0 International License (http://creativecommons.org/licenses/by/4.0/).

Recent investigation of BRB in MCI and AD patients by Shi et al. (2020b) has revealed early and progressive retinal vascular PDGFRβ deficiency and pericyte loss associated with retinal vascular Aβ_40_ and Aβ_42_ deposition in postmortem tissues from MCI and AD patients (Figures 1D–J). In a subset of patients with neuropathological reports, retinal vascular PDGFRβ expression significantly correlated with CAA and cognitive decline assessed by the Mini-Mental State Examination (MMSE). These data suggest that pericyte loss or PDGFRβ downregulation may precede AD progression. The retinal pericytes in cognitively impaired patients were found to accumulate Aβ_40_ and Aβ_42_ and undergo apoptosis, demonstrated by terminal deoxynucleotidyl transferase dUTP nick end labeling (TUNEL) assay and cleaved caspase-3 nuclear staining. Interestingly, a previous study detected increased neuronal apoptosis in the rat retina induced by intra-vitreous injection of Aβ_1–42_ oligomers (Fisichella et al., 2016). In a subsequent study, the Koronyo-Hamaoui group further discovered significantly augmented capillary degeneration in 8-month-old APP_SWE_PS1_ΔE9_ mice compared to wild type littermates that was further exacerbated in 12-month-old mice (Figures 2A,B; Shi et al., 2020a). Retinal capillary loss was associated with increased retinal vascular amyloidosis, indicating more BRB damage may be driven by vascular Aβ deposition and implicated in AD pathology (Shi et al., 2020a). Western blot analysis of whole retinal lysates revealed altered expression of key TJ molecules of the BRB, including claudin-1 and zonula occuludens-1 (ZO-1) (Figures 2C,D). These changes were also accompanied by elevated NF-κB p65 phosphorylation in retinas of 12-month-old ADtg mice, implicating upregulated inflammation in the retina with increased vascular amyloidosis burden. Having found these changes in retinal blood vessels and capillaries of AD-model mice, the authors sought to explore how these vascular pathologies may have affected BRB permeability. *In vivo* fluorescein (332 Da) imaging of APP_SWE_PS1_ΔE9_ mice showed live retinal vascular leakage in 12-month-old but not in 8-month-old mouse models of AD (Figure 2E). Intriguingly, intravenous injection of larger FITC-dextran (1,000 kDa) and Texas-Red-dextran (3 kDa) molecules in 6-month-old APP_SWE_PS1_ΔE9_ mice followed by *ex vivo* postmortem retinal imaging and quantification of the fluorescent signal indicated a dramatic increase in retinal vascular leakage of both molecules (Figures 2F,G). These BRB permeability changes in transgenic AD mice occur even earlier than the respective cerebral leakage measured by the same molecules (Lahiri et al., 2019). The difference between *in vivo* and *ex vivo* observations is suggestive of a shift in molecular size-dependent transporting mechanisms through the BRB in the AD transgenic mice model. Accordingly, a recent study utilizing the C57BL/6 mouse revealed a decrease in plasma protein transport activity through the BBB in the aged brain, driven by transport shifting from ligand-specific receptor-mediated to non-specific caveolar transcytosis (Yang et al., 2020). Whether this also occurs in AD patients’ BRB needs further validation. Overall, such discoveries have suggested that several BRB compartments are affected in AD disease progression that should be further evaluated as biomarkers for AD diagnosis.

### Cerebral Imaging for AD

Recent developments in brain imaging modalities have significantly improved the ability to rule-in AD related cerebral pathologies in at-risk populations (Johnson et al., 2012). These include MRI (fMRI) (Smith et al., 1999; Machulda et al., 2003; Dickerson et al., 2004; Johnson et al., 2006, 2012), fluorodeoxyglucose (FDG) positron emission tomography (PET) (Foster et al., 1983; Hoffman et al., 2000; Engler et al., 2006), amyloid PET imaging (Drzezga et al., 2008; Ikonomovic et al., 2008), PET imaging of copper trafficking (Torres et al., 2016; Andreozzi et al., 2017), and transcranial Doppler (TCD) ultrasound (Roher et al., 2011). However, these techniques are still subject to a variety of limitations such as high cost, low availability, low spatial resolution, low specificity, or involving the use of unsafe radio isotopes (Johnson et al., 2012). Nevertheless, current imaging techniques do not provide a solution for large scale screening of pre-symptomatic at-risk populations, which is the main goal of current efforts to develop more sensitive ocular examination techniques for AD diagnosis.

### Retinal OCT and OCT-A Imaging in MCI and AD Patients

Optical coherence tomography has been a pioneer technology in capturing retinal structural changes in living AD patients. This technology utilizes low-coherence light to acquire two- and three-dimensional images of retinal cross-sectional anatomy with micrometer resolution (Frohman et al., 2008; Popescu et al., 2011; Aumann et al., 2019). It provides non-invasive live measurements of retinal layer structure and is widely used in ophthalmic exanimations for diagnosis of glaucoma, age-related macular degeneration (AMD), diabetic retinopathy (DR), as well as other ocular diseases (Lang, 2007; Medical Advisory, 2009; Sathyan et al., 2012). Parisi et al. (2001) utilized this technology for the first time in AD patients, demonstrating a significant reduction in retinal nerve fiber layer (NFL) thickness as compared to healthy control individuals. Paquet et al. (2007) further described a significant reduction of retinal NFL thickness in MCI, mild AD, moderate AD, and severe AD patients compared to healthy controls. Subsequently, numerous studies verified these early studies and reported decreases in NFL, ganglion cell layer (GCL), and macula thickness correlating with cognitive decline (Kromer et al., 2014; Cunha et al., 2016; Doustar et al., 2017; Ferrari et al., 2017; Polans et al., 2017; Polo et al., 2017; Bulut et al., 2018; Janez-Escalada et al., 2019; Salobrar-Garcia et al., 2019; Czako et al., 2020; Dumitrascu and Koronyo-Hamaoui, 2020; Mejia-Vergara et al., 2021; Yan et al., 2021). OCT-adaptive optics is a relatively newer advancement of this technology which provides ultra-high-resolution images, including of blood vessel walls, that warrants further testing in the AD retina (Snyder et al., 2021).

Among the many advances in OCT technology, OCT-angiography (OCTA) has been specifically developed for the investigation of retinal blood vessels, revolutionizing the diagnosis of retinal vascular-related disorders (de Carlo et al., 2015; Chalam and Sambhav, 2016; Hagag et al., 2017). It provides high-resolution motion-contrast images based on backscattered light from neuronal and vascular tissues in the retina (Kashani et al., 2017). This enables visualization of various retinal vascular abnormalities such as microaneurysms, neovascularization, retinal vascular non-perfusion, reduced vascular density, and modified foveal avascular zone (FAZ) (Kashani et al., 2017). OCT-A received FDA approval in 2016 and has been rigorously used in diagnosis of retinal vascular diseases including DR, uveitis, AMD, and others (Pichi et al., 2017; Khadamy et al., 2018; Schneider and Fowler, 2018; Tey et al., 2019). The significant potential of this technology has recently led to a surge of research activity related to its utility in exploring retinal biomarkers in AD. An early case-control study by Bulut et al. (2018) on a total of 52 AD patients and healthy controls described a significant decrease in retinal vascular density, reduced retinal and choroidal thickness, as well as enlarged FAZ area in the patients. Shortly after, Jiang et al. (2018) based on 52 participants demonstrated lower densities of retinal vascular network, superficial vascular plexus (SVP), and deep vascular plexus (DCP) in MCI and AD patients, while O’Bryhim et al. (2018) with 32 participants validated increased FAZ area in AD patients. To date, such OCTA case-controlled studies seem to be largely consistent in demonstrating retinal vascular density loss and increased FAZ area in AD patients but differ in identifying vascular areas affected, the superficial vs. deep, or parafoveal vs. perifoveal vessels (Lahme et al., 2018; Sadda et al., 2019; Yoon et al., 2019; Zabel et al., 2019; Zhang et al., 2019; Czako et al., 2020; Lee J.Y. et al., 2020; Wu et al., 2020; Rifai et al., 2021). Overall, these are indeed breakthrough findings that warrant further investigation, considering OCTA is a relatively new technology. It is also important to note that sample sizes in most of these studies are relatively small. To better evaluate OCTA as a diagnostic tool for AD, longitudinal studies with a standardized consistent protocol and large case numbers are needed.

Blood–retinal barrier permeability in laboratory animals is usually measured by injecting fluorescent dyes such as fluorescein (Do carmo et al., 1998) or Evans blue (Xu et al., 2001), followed by *in vivo* or *ex vivo* imaging for retinal vascular leakage. Fundus fluorescein angiography (FFA) was developed based on visualizing fluorescent dye by fundus camera that has been widely used to evaluate retinal vascular circulation and BRB integrity (Marmor and Ravin, 2011). Another modified OCT method, OCT-leakage, was recently developed to monitor retinal edema, thus evaluating BRB damage (Cunha-Vaz et al., 2016; Cunha-Vaz, 2017). This method applies a proprietary algorithm to identify sites of decreased optical reflectivity, then the system quantifies and detects the correlation of retinal extracellular space. The developer tested OCT-leakage on 28 patients and provided consistent output between FFA and OCT-leakage for BRB damage in diabetic retinopathy (Cunha-Vaz et al., 2017). Both FFA and OCT-leakage can potentially be tested in cognitively impaired patients to investigate the potential of BRB permeability monitoring for AD diagnosis.

## Conclusion

In summary, recent advancements in retinal vascular research in AD patients and animal models have provided many potential candidate targets for non-invasive diagnosis by retinal vascular imaging. These include but are not limited to retinal vascular amyloidosis, FAZ area, vascular leakage, vascular blood flow and perfusion, TJ alteration, vascular density, pericyte and PDGFRβ loss, vascular branching complexity and others. Reports suggest that certain vascular abnormalities occur very early during AD progression and may predict cognitive decline in patients; thus, their detection may be critical for early diagnosis and prognosis prediction. However, since some of these vascular findings are commonly observed in retinal degenerative and inflammatory diseases, it is important to also consider AD-specific hallmark biomarkers such as Aβ and (p)tau for accurate diagnosis. Finally, with the recent development of retinal amyloid imaging (Koronyo et al., 2017; Dumitrascu et al., 2020; Ngolab et al., 2021), pericyte imaging (Schallek et al., 2013), OCTA and OCT-leakage (Cunha-Vaz et al., 2016; Cunha-Vaz, 2017), hyperspectral imaging (Hadoux et al., 2019; More et al., 2019; Lemmens et al., 2020), and FFA (Marmor and Ravin, 2011), future studies may pave a the way for next-generation non-invasive ophthalmic imaging technologies to facilitate AD monitoring and diagnosis.

## Author Contributions

HS and MK-H: draft manuscript and figures preparation. MK-H, HS, YK, AR, D-TF, NM, JS, and KB: manuscript editing. MK-H: study supervision. All authors read and approved the submitted version.

## Conflict of Interest

YK, MK-H, and KB are co-founders and stockholders of NeuroVision Imaging, Inc., Sacramento, CA, United States. MK-H, HS, YK, and KB are inventors on Patent Application No. 62/970,083 filed February 4, 2020 entitled “Method of Detecting Cognitive Impairment.” The remaining authors declare that the research was conducted in the absence of any commercial or financial relationships that could be construed as a potential conflict of interest.

## Publisher’s Note

All claims expressed in this article are solely those of the authors and do not necessarily represent those of their affiliated organizations, or those of the publisher, the editors and the reviewers. Any product that may be evaluated in this article, or claim that may be made by its manufacturer, is not guaranteed or endorsed by the publisher.
